# Control of litchi downy blight by zeamines produced by *Dickeya zeae*

**DOI:** 10.1038/srep15719

**Published:** 2015-10-26

**Authors:** Lisheng Liao, Jianuan Zhou, Huishan Wang, Fei He, Shiyin Liu, Zide Jiang, Shaohua Chen, Lian-Hui Zhang

**Affiliations:** 1 Guangdong Province Key Laboratory of Microbial Signals and Disease Control, South China Agricultural University, Guangzhou 510642, Peoples’ Republic of China; 2 Guangdong Innovative and Entepreneurial Research Team of Sociomicrobiology Basic Science and Frontier Technology, South China Agricultural University, Guangzhou 510642, China; 3 Institute of Molecular and Cell Biology, Agency for Science, Technology and Research (A*STAR), 61 Biopolis Drive, Proteos, Singapore 138673, Republic of Singapore; 4 Department of Biological Sciences, National University of Singapore, Republic of Singapore

## Abstract

Zeamines (ZMS), a class of polyamine-polyketide-nonribosomal peptide produced by bacterial isolate *Dickeya zeae*, were shown recently to be potent antibiotics against some bacterial pathogens. In this study, the results indicated that ZMS showed antifungal activity against *Peronophythora litchii* and other fungal pathogens. The activity of ZMS against the oomycete pathogen *P. litchi*, which causes the devastating litchi downy blight, was further investigated under *in vitro* and *in vivo* conditions. ZMS displayed potent inhibitory activity against the mycelial growth and sporangia germination of *P. litchii*. At a concentration of 2 μg/mL, about 99% of the sporangia germination was inhibited. Scanning electron microscopy and transmission electron microscopy analyses showed that treatment with ZMS could cause substantial damages to the oomycete endomembrane system. Furthermore, treatment of litchi fruits with ZMS solution significantly (*P* < 0.05) reduced the fruits decay and peel browning caused by *P. litchii* infection during storage at 28 °C. Taken together, our results provide useful clues on the antifungal mechanisms of ZMS, and highlight the promising potentials of ZMS as a fungicide, which in particular, may be useful for prevention and control of litchi fruits decay and browning caused by *P. litchii* infection during storage and transportation.

*Peronophythora litchii*, an oomycete pathogen, is a transitional species between *Phytophthora* and *Peronospora*, which causes devastating downy blight on litchi in the subtropical regions of southern China, India, Thailand, and Vietnam^1^,^2^,^3^,^4^. The pathogen causes panicle rot, withering and fruit decay, resulting in significant postharvest losses in Southern China and more than 60% of commercial losses have been reported^5^,^6^. *P. litchii* can grow on artificial media, which is unique as most downy mildews cannot be cultured under *in vitro* conditions^2^,^5^. In order to control the diseases of postharvest litchi fruits, a wide range of chemical fungicides have been tested and evaluated^7^. Given the unavoidable emergence of fungicide resistance as well as toxicological risks and environmental pollution concerns^8^, it is important to discover and develop alternative means to prevent and control postharvest litchi.

Zeamines (ZMS), including zeamine and zeamine II, are recently discovered novel antibiotics, which were initially identified from some isolates of *Dickeya zeae*^9^,^10^,^11^. Zeamine II is a long chain aminated polyketide, and zeamine contains the same polyketide structure as zeamine II with an extra valine derivative moiety (Fig. 1). In addition to *D. zeae*, a bacterial isolate *Serratia plymuthica* RVH1 was also shown to produce ZMS^12^,^13^. Polyketides are important class of microbial natural products, which possess potent bioactivities and many of them have been successfully used as antibiotics, immunity inhibitors, antitumor drugs and antifungals^14^,^15^,^16^. We showed previously that ZMS are potent antibiotics against a few bacterial pathogens of medical importance including multidrug-resistant bacteria such as *Staphylococcus aureus* and *Pseudomonas aeruginosa*^9^,^10^. ZMS inhibited the growth of both Gram-positive and Gram-negative bacteria at a low concentration, suggesting that their bioactivity is worthy of further exploration^9^,^12^,^13^.

In this study, we determined the ZMS activity against *P. litchii* by *in vitro* and *in vivo* tests. The effects of the treatments on oomycete cells were also evaluated by scanning electron microscopy (SEM), transmission electron microscopy (TEM) and ion leakage analysis. Our results provide useful insights into the mechanism of ZMS against oomycete pathogens and may also present a useful solution to reduce economic losses of litchi fruits caused by *P. litchii* and other fungal pathogens.

## Results

### ZMS are potent antibiotics against bacterial and fungal pathogens

The results showed that all the bacterial pathogens were highly sensitive as similar to the sensitivity of *Escherichia coli* DH5α (showing high sensitivity to antibiotic) to ZMS, with minimum inhibitory concentration (MIC) values ranging from 0.5–4 μg/mL (Table 1). ZMS also showed excellent inhibitory activity against 6 fungal and oomycete pathogens tested, i.e., *Rhizoctonia solani*, *Pyricularia oryzae* Cav, *Colletotrichum gloeosporioides*, *P. litchii*, *Fusarium oxysporum*, and *Botrytis cinerea*, with MIC values varying between 8–50 μg/mL (Table 1). Among the oomycete and fungal pathogens tested for MIC, the litchi oomycete pathogen *P. litchi* was the most sensitive to ZMS.

### Inhibition of mycelial growth and sporangia germination

**Figure 2:**
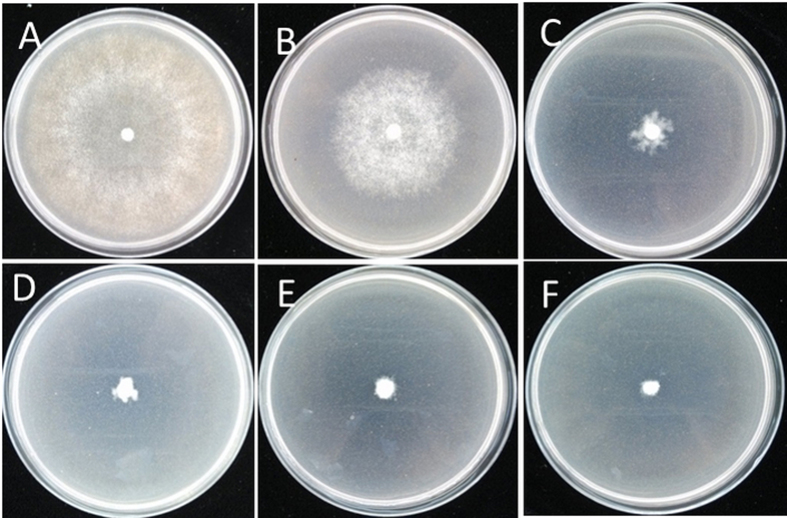
ZMS inhibited mycelial growth of *P. litchii in vitro*. (**A**) Control; (**B**) 0.5 μg/mL ZMS; (**C**) 1 μg/mL ZMS; (**D**) 2 μg/mL ZMS; (**E**) 4 μg/mL ZMS and (**F**) 8 μg/mL ZMS.

**Figure 3:**
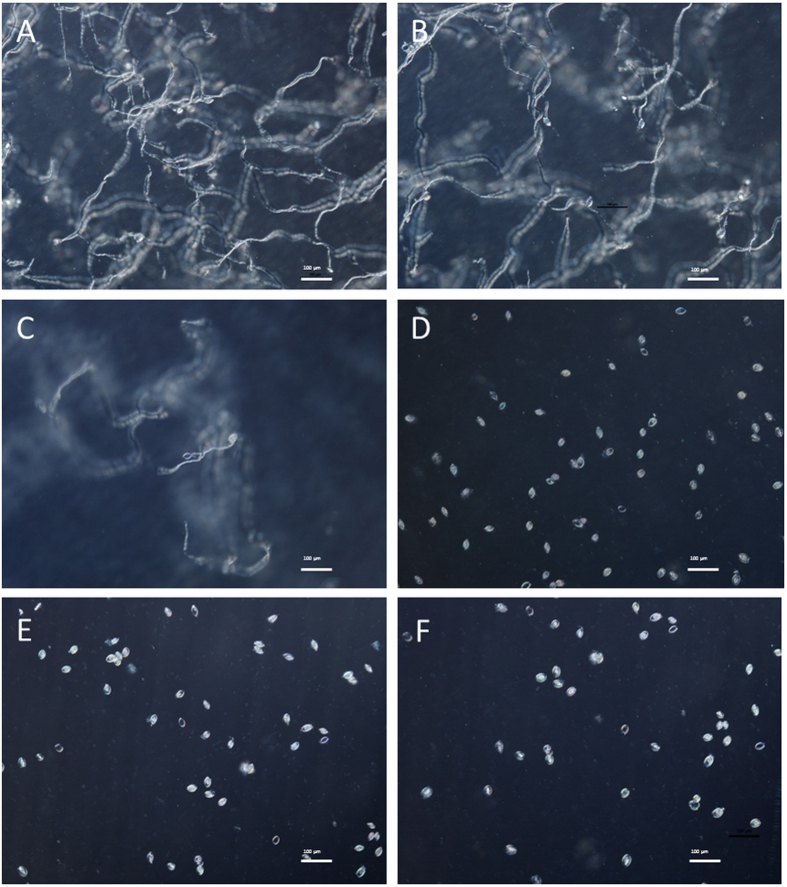
ZMS inhibited sporangia germination of *P. litchii in vitro*. (**A**) Control; (**B**) 0.5 μg/mL ZMS; (**C**) 1 μg/mL ZMS; (**D**) 2 μg/mL ZMS; (**E**) 4 μg/mL ZMS and (**F**) 8 μg/mL ZMS.

ZMS significantly inhibited the mycelial growth and sporangia germination of *P. litchii* (Figs 2 and 3). At 4 μg/mL, ZMS significantly (*P* < 0.05) inhibited mycelial growth. Similarly, at a concentration of 2 μg/mL ZMS, germination of up to 99% sporangia was inhibited. Further observation by light microscopy indicated that application of 2 μg/mL ZMS prevented sporangia germination and release of zoospores (Fig. 3).

### ZMS treatment changes the morphologies and ultrastructures of *P. litchii*

**Figure 4:**
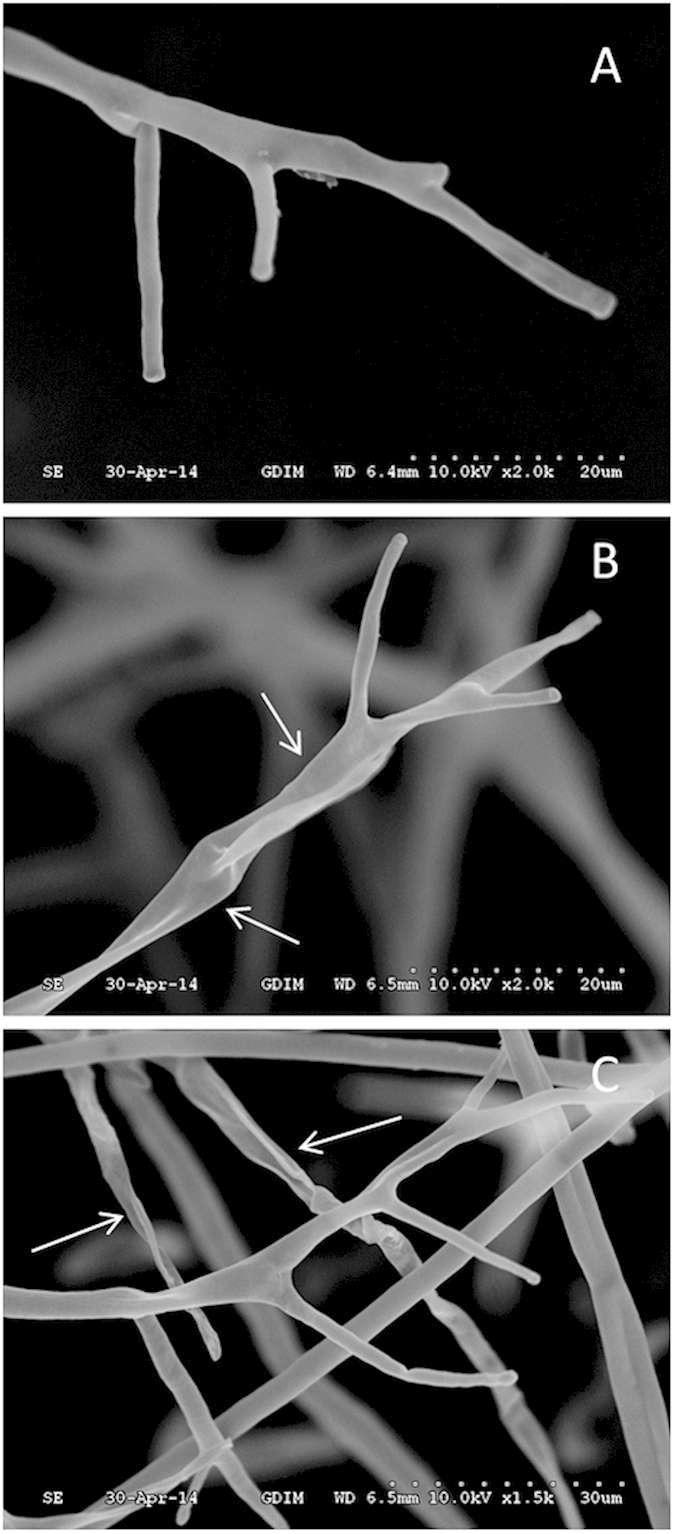
Scanning electron micrographs for the hyphae of *P. litchii* grown for 5 days on CAM plates with or without ZMS at 25 °C. (**A**) control; (**B**,**C**) 2 μg/mL ZMS. Arrow indicates shrinking or distorted oomycete cells.

To understand the mechanism of inhibition, the effect of ZMS on the morphology of *P. litchii* hypha were examined by scanning electron microscopy (SEM) (Fig. 4). The ZMS-untreated control mycelia grown on CAM without ZMS showed normal morphology with linear, regular and uniform cell walls (Fig. 4A). In contrast, ZMS treatment resulted in shrinking and distorted hypha with distinct craters on the cell walls (Fig. 4B,C).

**Figure 5:**
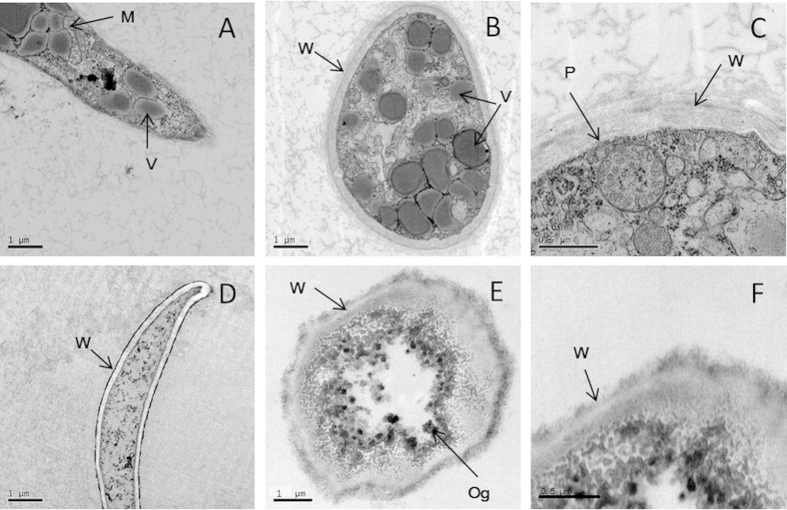
Transmission electron micrographs for the hyphae of *P. litchii* grown for 5 days on CAM plates with or without ZMS at 25 °C. (**A–C**) control; (**D–F**) 2 μg/mL ZMS; (**A,D**) longitudinal section through the hyphae of *P. litchii* ( × 12,000); (**B,E**) tangential section through the hyphae ( × 12,000); (**C,F**) cell wall and plasma membrane of the hyphae ( × 20,000). M, mitochondria; P, plasma membrane; V, vacuoles; and W, cell wall.

Transmission electron microscopy (TEM) analysis showed that in contrast to the ZMS-untreated control (Fig. 5A–C), the ZMS treatment led to thickened oomycete cell walls (Fig. 5D), and damaged cell walls and plasma membranes (Fig. 5E,F). In addition, the membranes of mitochondria, vacuoles and organelles were injured or even destroyed by ZMS application (Fig. 5D,E). Moreover, intracellular components in the ZMS-treated hyphae were indistinct (Fig. 5D,F).

### ZMS treatment increases hyphal ion leakage analysis

**Figure 6:**
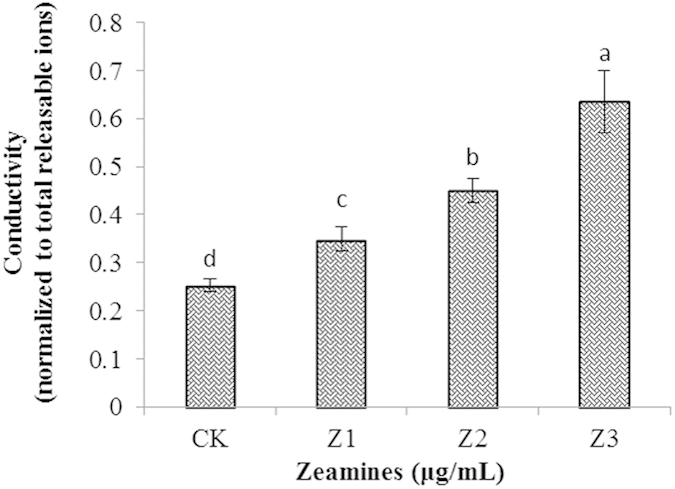
Analysis of ion leakage of *P. litchii* by treatment with ZMS after 5 days at 25 °C. CK, control; Z1, 0.5 μg/mL ZMS; Z2, 1 μg/mL ZMS; Z3, 2 μg/mL ZMS. Data are the means from three replicates per treatment with five plates in each replicate.

Microscopic data show that ZMS treatment might affect the integrity of oomycete cell membranes. Therefore, we further determined the ion leakage of oomycete cells which were treated with and without ZMS. To this end, for each treatment we collected 10 hypha disks from 5 plates by using a 4-mm puncher; and the hypha cells were washed to dilute ion concentration at the cut edges. The results showed that ZMS treatment significantly (*P* < 0.05) increased conductivity of oomycete hypha cells (Fig. 6). When treated with a low concentration of ZMS at 0.5 μg/mL, the oomycete hypha cells conductivity was increased by over 35%. When ZMS concentration was increased to 2 μg/mL, the oomycete hypha cells conductivity was elevated by about 140% over the control (Fig. 6). These results were consistent with the previously observed damages of ZMS on oomycete cell membrane (Fig. 5), application of ZMS could cause severe ion leakage in the hyphal cells of *P. litchii.*

### Effect of ZMS on litchi fruit decay and browning caused by inoculation with *P. litchii*

**Figure 7:**
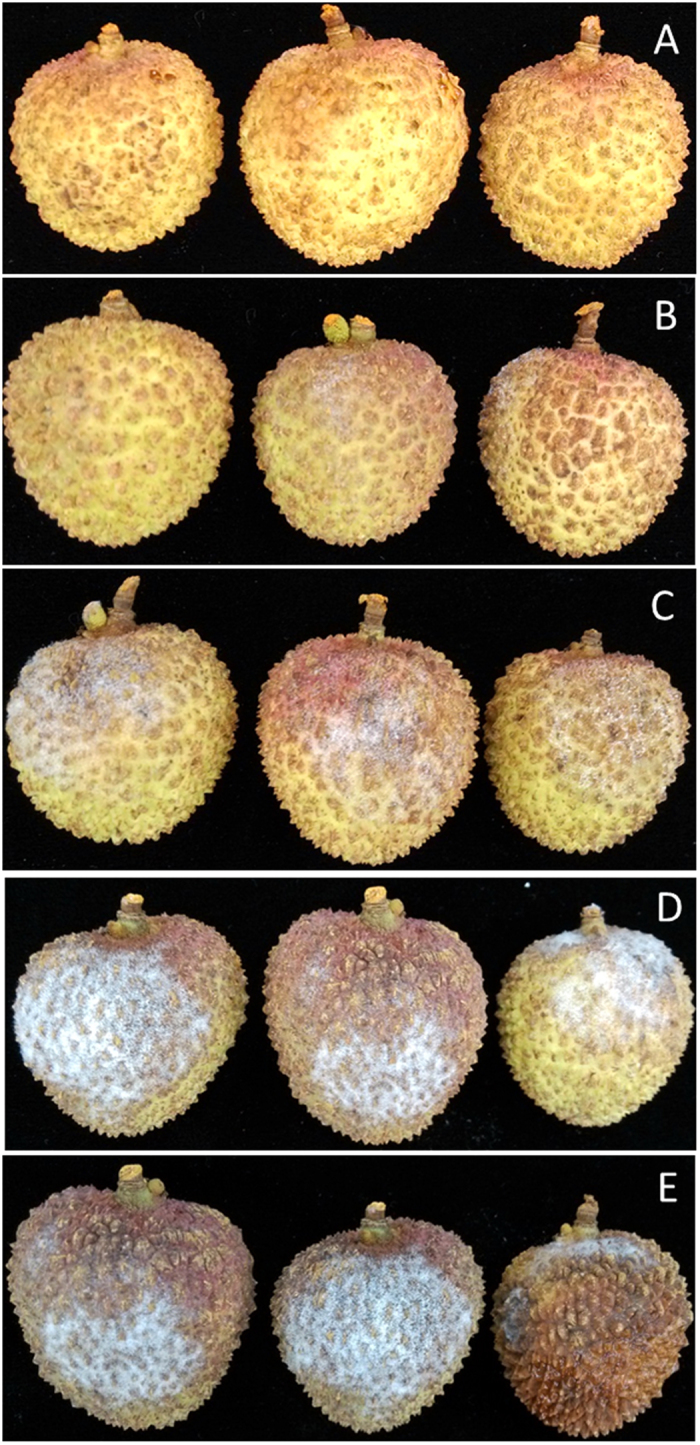
Effect of ZMS for reducing litchi disease caused by *P. litchii* after 5 days of storage at 28 °C. (**A**) Control (without inoculation); (**B**) 8 μg/mL ZMS; (**C**) 4 μg/mL ZMS; (**D**) 2 μg/mL ZMS; (**E**) without ZMS. Each treatment consisted of three replicates with 60 fruits in each replicate.

**Figure 8:**
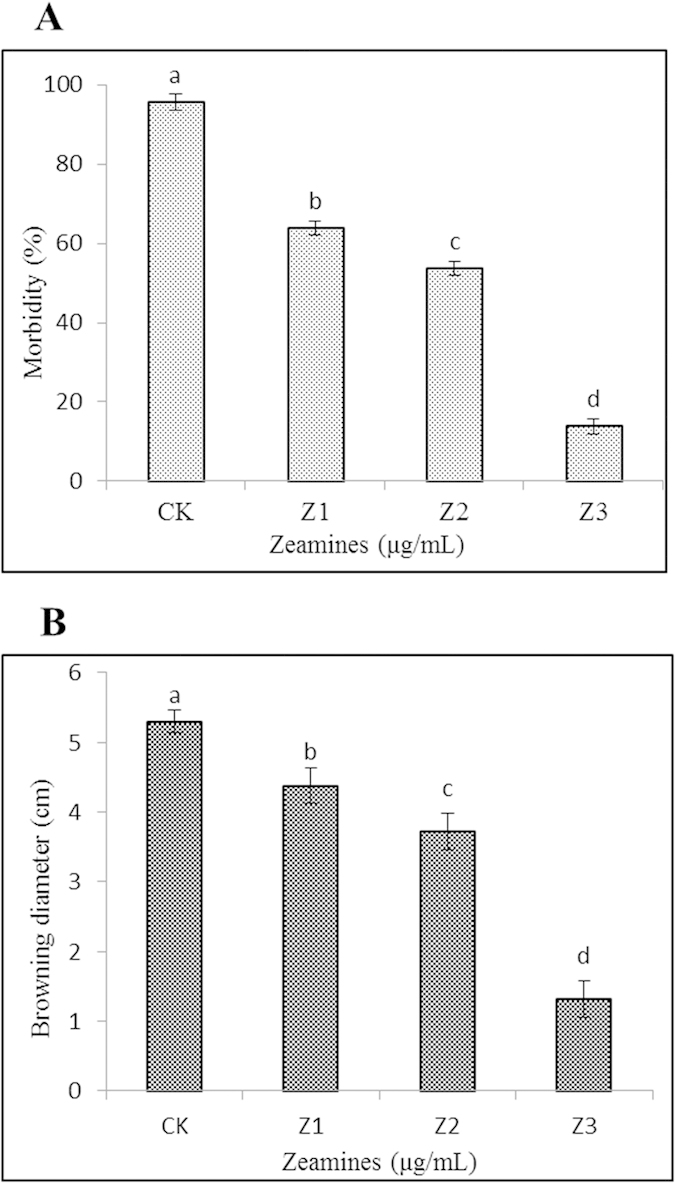
Effect of different concentrations of ZMS on morbidity (A) and browning diameter (B) of harvested litchi fruits inoculated with *P. litchii* after 5 days of storage at 25 °C. Data are the means from three replicates per treatment.

To evaluate the potential of ZMS in control and prevention of oomycete infection on litchi fruits, the newly collected fruits were treated with various concentrations of ZMS before inoculation of *P. litchii.* After 5-days at 28 °C, the disease severities of ZMS-treated fruits and water-treated controls were determined. The results showed that application of ZMS substantially reduced the disease severity, especially at higher ZMS concentrations (Fig. 7). Litchi fruits with ZMS at 2, 4, 8 μg/mL reduced the disease morbidity by 37.2%, 49.7% and 86.2%, respectively, in comparison with the noninoculated controls (Fig. 8A).

In addition, peel browning of postharvest litchi fruits caused by *P. litchii* is one of the major factors affecting the commercial value of litchi fruits. Treatment with ZMS substantially reduced fruit peel browning (Fig. 8B). After storage at 28 °C for 5 days, the browning diameter of the fruits treated with 2, 4 and 8 μg/mL ZMS was about 4.4, 3.7 and 1.3 cm, respectively (Fig. 8B), whereas the control was 5.3 cm. ZMS treatment did not show any visible effect on the color and taste of litchi fruit meat compared with the control. The results suggested that ZMS treatment can effectively prevent and control *P. litchii* infection on litchi fruits.

## Discussion

Litchi downy blight is important postharvest disease caused by *P. litchii*, which leads to considerable losses during storage and transportation^17^,^18^. The disease is managed principally by application of synthetic fungicides such as mancozeb, cymoxanil, sulfur dioxide and benomyl^7^,^17^. To deal with the unavoidable emergence of fungicide tolerance and resistance problems, it is critical to discover and identify new antifungal molecules with different mechanisms for the control of this disease^6^,^7^,^19^. In this study, we showed for the first time that ZMS, a new type of antibiotics produced by *Dickeya zeae* EC1, can effectively suppress the decay of post-harvest litchi fruits and peel browning caused by *P. litchii*. We also presented evidence that the antifungal activity of ZMS is associated with a profoundly destructive effect on oomycete cell membrane integrity.

The results from this study indicate that ZMS has potent activity against *P. litchii* sporangia germination and mycelial growth *in vitro*. Supplementation of 2 μg/mL ZMS in the culture medium led to 97.5% and 99% inhibition on *P. litchii* mycelial growth and sporangia germination, respectively. In contrast, soaking litchi fruits with 8 μg/mL ZMS solution for 20 min reduced fruit decay and peel browning by about 86% and 78%, respectively. The results showed that a large amount but not all of ZMS molecules in the soaking solution could be absorbed or attached to the peels of litchi fruits. A thorough formulation study by including comparable adjuvants to improve ZMS absorbance to litchi fruits may substantially increase the efficacy of ZMS in control and prevention of litchi downy blight.

The results from this study also provide clues on how ZMS act to inhibit oomycete growth and development. SEM analysis showed that ZMS treatment caused deformation and concave collapses of the hypha of *P. litchii*. Consistent with the SEM findings, TEM analysis found that the plasma membranes of the hypha were damaged severely and certain intracellular organelles disappeared. Moreover, ZMS treatment resulted in ion leakage of hyphal cells. These data suggested that ZMS target cell membranes and affect the membrane permeability. ZMS are typical cationic molecules with four positively charged amino group substitutions evenly distributed in the conserved 40-carbon hydrophobic alkane chain^9^,^10^. These chemical properties suggested that ZMS may interact with the negatively charged membrane components of the oomycete cells to influence membrane permeability, in a mechanism similar to cationic antimicrobial peptides^20^,^21^,^22^. Zeamines also caused bacterial membrane lysis at a lethal concentration and resulted in “fast-killing” of *Caenorhabditis elegans*^23^,^24^. *P. litchii* is more sensitive to ZMS compared to the fungal pathogens used in this study. Oomycetes differ from true fungi in their cell wall composition which account for their different ZMS sensitivity observed in our study^2^,^3^,^5^. Hence together, these findings have established a molecular mechanism with which ZMS act as potent and wide spectrum antibiotics against bacterial, nematode, and oomycete pathogens^9^,^10^,^24^.

Interestingly, in contrasting to their potent activity against bacterial pathogens and *P. litchii* (Table 1), zeamines are relatively weak phytotoxins against rice seed germination. Even for the most sensitive rice variety we have tested, rice seeds germination was not inhibited by 60 μM of either zeamine or zeamine II (equivalent to 39 μg/mL zeamine II or 50 μg/mL zeamine)^10^. Similarly, we found that treatment of litchi fruits with 8 μg/mL ZMS alone did not cause any visible or detectable changes in the color and taste of litchi fruit. This apparent desirable selectivity may facilitate further development of ZMS as a useful antifungal solution for practical control and prevention of postharvest litchi fruit diseases.

In conclusion, ZMS showed an excellent activity against oomycete *P. litchii* in agreement with previous findings on its bactericidal and fungicidal activity. We showed that treatment with ZMS could cause substantial ultrastructural changes of *P. litchii* cells, including distortion of cell walls and lysis of endomembrane systems, particularly the plasma membrane, mitochondria, and vacuoles. We also found that ZMS could be effectively used to control postharvest litchi fruit decay and browning caused by *P. litchii*. Moreover, ZMS treatment did not show any visible effect on the color and taste of litchi fruit compared with the untreated control. Our results provide useful clues on the antifungal mechanisms of ZMS, and highlight the promising potentials of ZMS as a fungicide, which in particular, may be useful for prevention and control of litchi fruits decay and browning caused by *P. litchii* infection during storage and transportation.

## Materials and Methods

### Microorganisms and culture conditions

A wild-type strain *P. litchii* was isolated from a diseased fruit in an orchard of the Shenzhen city, China. A single sporangium was isolated from a single sporangiophore of this strain by a sterile needle. *P. litchii* was grown and maintained on carrot agar medium (CAM, consisting of 100.0 g/L carrot slices squeezed by juice extractor, 16.0 g/L agar) in an incubator at 25 °C in the dark. All the fungal species (Table 1) were provided by Dr. Zide Jiang from the Laboratory of Mycology, South China Agricultural University, Guangzhou, P. R. China. The fungal strains were grown on potato dextrose agar (PDA, consisting of 200.0 g/L sliced potatoes boiled for 30 min, 20.0 g/L dextrose, 20.0 g/L agar) at 28 °C. *E. coli* DH5α was routinely grown in Lysogeny broth (LB, consisting of 10.0 g/L Bacto tryptone, 5.0 g/L yeast extract, and 10.0 g/L NaCl, pH 7.0) at 37 °C. The other bacterial species were maintained in LB medium at 28 °C. *D. zeae* strain EC1 was grown in LS5 medium (consisting of 9.25 g K_2_HPO_4_, 3.3 g/L KH_2_PO_4_, 1.4 g/L NH_4_NO_3_, 12.7 g/L sucrose, 1 g/L KCl, 1 g/L asparaginate, and 0.25 g/L MgSO_4_, pH 7.0) at 28 °C for preparation of ZMS^25^.

### ZMS preparation

ZMS purification was conducted according to our previously described method with minor modifications^10^,^11^. Briefly, strain EC1 was grown in LS5 medium for 48 h, and the bacterial supernatants were extracted by equal volume of n-butyl alcohol three times. The organic phases were collected and combined, and n-butyl alcohol was removed by rotary evaporator. The active crude extract in the residues, dissolved in organic solvent (methanol/chloroform = 1:1), was subjected to gel filtration chromatography on Sephadex LH-20, with organic solvent (methanol/chloroform = 1:1) as the eluting solvent. The active fractions were concentrated by rotary evaporator and dissolved in methanol for separation by using both semipreparative and high performance liquid chromatography (HPLC). The ZMS fractions were collected for bioassay of antimicrobial activities, as described previously^10^,^11^.

### MIC determination

Minimum inhibitory concentration (MIC) is the lowest concentration of an antimicrobial that inhibits the visible growth of a microorganism after overnight incubation^26^. For determination of MIC against bacterial pathogens, a standard protocol was followed^9^,^26^. In brief, ZMS was prepared as a 10% methanol stock solution, which was diluted with 10% methanol to give serial two-fold dilutions. Then, the dilutions were added into 96-well plates containing standard concentration of testing bacteria using *E. coli* strain DH5α as a control. After 24 h incubation at 28 °C, the plates were collected to measure OD_600_. For determination of MIC against oomycete and fungal pathogens, the experiment was carried out by using the agar dilution method^27^. Briefly, ZMS was diluted to prepare serial ten-fold dilutions, which were then added into 55 °C CAM and PDA solid medium, respectively. oomycete and fungal strains were inoculated respectively, and MICs were determined after 5 days incubation at 25 °C. The experiment was repeated twice with triplicate.

### Fruit material

Litchi fruits were harvested from an orchard in Shenzhen city, China. The fruits were selected for uniform shape, color and size as well as disease-free. Before the treatment and inoculation, the fruits were random divided into five groups and 60 fruits in each group for the experiments *in vivo*.

### Mycelial growth assay

Agar dilution assay was used to test the inhibitory effect of ZMS on mycelial growth of *P. litchii*. A mycelial agar plug (4 mm in diameter) was excised from 5-day-old colonies on CAM plates with a cork borer and placed mycelia upside down on CAM plates containing different concentrations of ZMS (0.5 to 8 μg/mL). Sterile distilled water was used as the control. The plates were incubated at 25 °C for 5 days. Three replicate plates were used for each treatment. The diameters of mycelial growth were recorded to assess the inhibitory effect of ZMS.

### Sporangia germination assay

The assay was conducted according to the described method with minor modifications^28^. Briefly, sporangia of *P. litchii* were collected from 5-day-old cultures on CAM media at 28 °C in the dark. The effect of ZMS on sporangia germination of *P. litchii* was assessed on CAM. Serial dilutions of ZMS were prepared in 10% methanol (methanol at this concentration has no effect on sporangia germination of *P. litchii*), which were then added into 55 °C CAM to prepare Petri dish plates (containing different concentrations of ZMS from 0.5 to 8 μg/mL). A 100 μL aliquot of sporangia suspension (1 × 10^6^ sporangia per mL) of *P. litchii* was spread to each Petri dish. After 24 h of incubation at 28 °C in dark, the germinated and non-germinated sporangia of each plate were observed and counted under a high-end microscope (Eclipse 90i imaging, Nikon). A sporangium was considered to be germinated when germ tube length was 1.5 times the sporangium diameter. The experiment was repeated twice and each treatment 200 sporangia per duplicate were observed.

### Scanning electron microscopy (SEM) observation

Following the described method^29^, the hypha or sporangia of *P. litchii* were observed by scanning electron microscopy (SEM). A 4-mm diameter disk of mycelium was taken from the periphery of the colony grown on CAM medium containing 2 μg/mL ZMS after 5 days incubation at 25 °C. The mycelium disk of colony grown on CAM medium without ZMS was used as control. The mycelium disks were permeated into 2.5% glutaraldehyde (prepared with 0.1 mol/L phosphate buffer, pH 7.2) at 4 °C overnight. The samples were post-fixed for 4 h in 1% OsO_4_ solution after rinsing with 0.1 mol/L phosphate buffer (pH 7.2) for 15 min 3 times. The fixed samples were dehydrated by rinsing separately with ethanol solutions of 10%, 30%, 50%, 70%, and 90%, for 10 min, followed by a three times rinsing with 95% and absolute ethanol for 15 min, respectively. After critical point drying under carbon dioxide and sputter coating with gold platinum, the sample was examined using a scanning electron microscope (S-3000N, Hitachi) operated at 25 kV.

### Transmission electron microscopy (TEM) observation

After taking 4-mm disk samples from the colony grown for 5 days on CAM medium containing 2 μg/mL ZMS, the samples were fixed with 2.5% glutaraldehyde at 4 °C 24 h, followed by post-fixation in 1% OsO_4_ solution at 4 °C overnight, after rinsing with 0.1 mol/L phosphate buffer (pH 7.2) for 15 min 3 times. As described above, the materials were dehydrated by ethanol solutions, finally embedded in epoxy media. Blocks were sectioned by ultramicrotome (EM UC7, Leica) into ultrathin sections about 70 nm. The ultrathin sections were stained with 2.5% lead citrate solution for 20 min and for 40 min with 2% uranyl acetate solution. Finally, the sections were observed using a TEM transmission electronmicroscope (Tecnai, FEI) at 100 kV.

### Measurement of ion leakage

To prepare oomycete samples following the described method with minor modification^30^, 10 pieces (12.56 mm^2^) of 5 days old mycelial agar plugs were taken using a 4-mm diameter punch. The samples were washed in MilliQ-purified water and dried on a filter paper, which were then put into a 20-ml syringe containing 10 ml of MilliQ-purified water. The samples were infiltrated with the water by applying a vacuum by depressing the plunger of syringe with the needle-end sealed. Repetitive infiltration combined with manual shaking was performed for 10 min. Liquid was then transferred to a sterile tube, and the sample conductivity (C_s_) was measured by using a conductivity meter (FE30–FiveEasy™, Mettler Toledo, Switzerland). Hypha samples were then taken from the syringe and were put into MilliQ-purified water. After boiling for 30 min, samples were cooled down to room temperature for 2 h and conductivity was read to determine total releasable ion levels (C_t_). Each treatment contained three replicates and the experiment was repeated at least five times. The conductivity value was derived by normalization of C_s_ to C_t_ readings and shown as mean plus standard error for three replicates.

### Effect of zeamines on infection by *P. litchii*

To assess the effect of ZMS on the control of litchi fruit oomycete infections, a previous described method was followed with minor modifications^19^,^28^. In brief, three groups of fresh litchi fruits were soaked in 2, 4, and 8 μg/mL of ZMS for 20 min, respectively, two groups were soaked in sterile water as control. A 2-mm deep and 4-mm wide cross-like wound was cut with a sterile scalpel on the equatorial area of each fruit peel. Subsequently, five groups (ZMS and sterile water treatment) of fruits were inoculated with 10 μL of sporangia suspension of *P. litchii* (1 × 10^6^ sporangium/mL) or equal volume of sterile water into the wound with a pipette. All fruits were transferred into a sterile square box and kept at 28 °C for 5 days. Each treatment consisted of three replicates with 60 fruits in each replicate.

### Assessment of fruit decay and peel browning

Decay development was monitored daily, and the severity scale of fruit decay was determined according to the extent of decay on the peel: 1 = no decay; 2 = slight decay; 3 = 25% decay; 4 = 25–50% decay; 5 ≥ 50% decay. The morbidity was calculated as: Σ(decay scale × proportion of fruits corresponding to each scale)/5 × 100%. The extent of peel browning was determined by measuring the diameter of browning area: 1 = no browning; 2 = slight browning; 3 ≤ 25% browning; 4 = 20–50% browning; 5 ≥ 50% browning (poor quality). The browning index was calculated with the following formula ∑(browning scale × proportion of corresponding fruit within each class)^18^.

### Statistical analysis

Data were expressed as mean ± standard error from three replicates per treatment. Differences among different treatments were determined by one-way analysis of variance (ANOVA) using Statistics Analysis System (SAS) version 9.0 at the 0.05 level.

## Additional Information

**How to cite this article**: Liao, L. *et al.* Control of litchi downy blight by zeamines produced by *Dickeya zeae*. *Sci. Rep.* **5**, 15719; doi: 10.1038/srep15719 (2015).

## Figures and Tables

**Figure 1:**
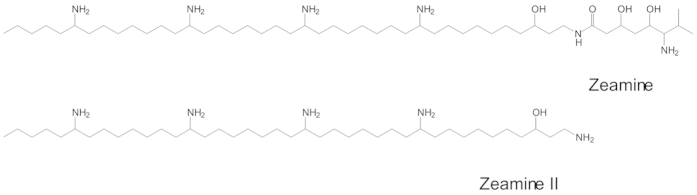
Chemical structures of zeamine and zeamine II.

**Table 1:** Minimal inhibitory concentration of ZMS on some pathogenic microorganisms.

	Microorganism	ZMS (μg/mL)
Bacteria	*Escherichia coli* DH5α	1
	*Pseudomanas solanacearum*	0.5
	*Xanthomonas oryzae*	0.5
	*Xanthomonas citri*	4
	*Dickeya dadantii*	4
	*Agrobacterium tumefaciens*	2
	*Erwinia chrysanthemi*	4
	*Acinetobacter johnsonii*	2
Fungi	*Rhizoctonia solani*	15
	*Pyricularia oryzae Cav*	50
	*Colletotrichum gloeosporioides*	40
	*Peronophythora litchii*	8
	*Fusarium oxysporum*	20
	*Botrytis cinerea*	40
